# SGLT2 inhibitor upregulates myocardial genes for oxidative phosphorylation and fatty acid metabolism in Gαq-mice

**DOI:** 10.1016/j.jmccpl.2025.100296

**Published:** 2025-04-09

**Authors:** Jordan M. Chambers, Dominique Croteau, David R. Pimentel, Adam C. Gower, Marcello Panagia, Tomas Baka, Fuzhong Qin, Ivan Luptak, Wilson S. Colucci

**Affiliations:** Cardiovascular Medicine Section and Myocardial Biology Unit, and the Clinical and Translational Institute, Boston University School of Medicine, Boston, MA, United States of America

**Keywords:** Sodium-glucose linked transporter 2 inhibitor, Cardiomyopathy, Heart failure with reduced ejection fraction, Cardiac metabolism, Mitochondria, Energetics

## Abstract

**Background:**

Mitochondrial dysfunction with decreased ATP production and increased release of reactive oxygen species (ROS) is a hallmark of the failing heart. Although SGLT2 inhibitors have been shown to improve myocardial metabolism in the failing heart, independent of diabetes, the effect on mitochondria is not clear.

**Objectives:**

Our goal was to test the effect of the SGLT2 inhibitor ertugliflozin on mitochondrial gene expression and function in myocardium and isolated mitochondria from non-diabetic mice with dilated cardiomyopathy due to cardiac-specific over-expression of Gαq.

**Methods:**

Gαq and wild type (WT) littermates 4 weeks of age were treated for 16 weeks with or without the SGLT2 inhibitor ertugliflozin (ERTU) formulated in the chow (0.5 mg/g chow).

**Results:**

From weeks 4 to 20, Gαq mice developed progressive cardiac hypertrophy, dilation, contractile dysfunction, myocyte apoptosis and interstitial fibrosis – all of which were prevented by ERTU treatment. Isolated cardiac mitochondria from Gαq mice had decreased maximal ATP production and increased ROS release - both of which were normalized by ERTU. In isolated beating hearts from Gαq mice, contractile reserve and high energy phosphates measured simultaneously by ^31^P NMR spectroscopy were decreased - and both were improved by ERTU. In Gαq mice, marked suppression of myocardial gene programs for oxidative phosphorylation and fatty acid metabolism was reversed by ERTU.

**Conclusions:**

The SGLT2 inhibitor ERTU corrected the expression of myocardial gene programs for oxidative phosphorylation and fatty acid metabolism, and was associated with increased production of ATP, decreased release of mitochondrial ROS, and amelioration of the consequences of mitochondrial dysfunction on myocardial structure and function.

## Highlights

• Mitochondrial dysfunction is a hallmark of the failing heart.
• Overexpression of Gαq in mice causes heart failure and mitochondrial dysfunction.
• Gαq mice were treated with the SGLT2 inhibitor ertugliflozin for 16 weeks.
• Ertugliflozin prevented structural remodeling and mitochondrial dysfunction.
• Ertugliflozin corrected adverse programing of cardiac metabolic genes.

## Graphical abstract

**Figure:**
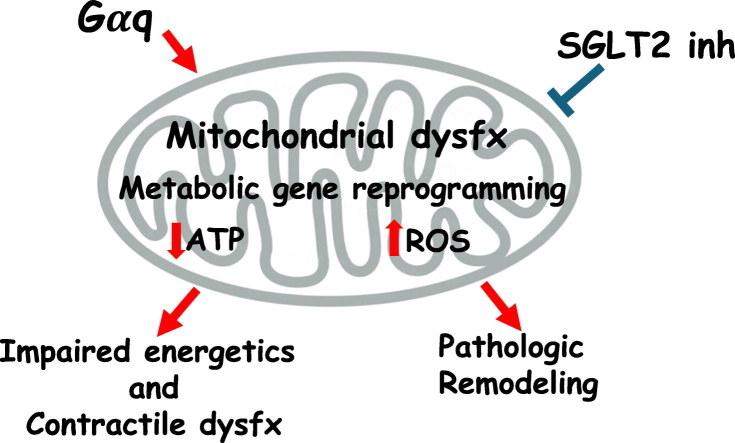

## Non-standard abbreviations and acronyms

SGLT2: sodium-glucose linked transporter 2
DCMP: diabetic cardiomyopathy
ERTU: ertugliflozin
NMR: nuclear magnetic resonance
OXPHOS: oxidative phosphorylation
FAM: fatty acid metabolism
LVH: left ventricular hypertrophy
ROS: reactive oxygen species
HOMA-IR: homeostasis model assessment of insulin resistance
4-HNE: 4-hydroxynonenal
KH: Krebs-Henseleit
LVDevP: left ventricular developed pressure
LVSP: left ventricular systolic pressure
LVEDP: left ventricular end diastolic pressure
PCr: phosphocreatine
Pi: inorganic phosphate
RPP: rate pressure product
GSEA: gene set enrichment analysis
GO: gene ontology
NES: normalized enrichment score
FDRq: false-discovery rate corrected *p*-value
EDD: end diastolic dimension
ESD: end systolic dimension
FS: fractional shortening
CSA: cross-sectional area

## 1 Introduction

In failing myocardium, mitochondrial dysfunction leads to impaired ATP production and elevated levels of reactive oxygen species (ROS) [1]. Decreased energy production contributes to abnormalities in ATP-dependent processes including, but not limited to, contraction and relaxation. Increased production and release of mitochondrial ROS plays a central role in mediating multiple aspects of structural and functional remodeling including myocyte hypertrophy, myocyte apoptosis, interstitial fibrosis and post-translational protein modification [2,3].

SGLT2 inhibitors have had a dramatic impact on the treatment of heart failure in the presence or absence of diabetes [4,5]. While it appears that these drugs improve cardiac structural and functional remodeling in the absence of diabetes [[6], [7], [8], [9]], the targets and mechanism(s) of action remain unclear [10,11]. Recently, we found that mice treated with a SGLT2 inhibitor demonstrated marked upregulation of cardiac metabolic genes that promote oxidative phosphorylation and fatty acid metabolism [12]. While the goal of that work was to determine the effect of SGLT2 inhibition in a model of diabetic cardiomyopathy caused by diet excess, a noteworthy finding was that pro-metabolic programming of the heart by the SGLT2 inhibitor also occurred in non-diabetic control mice, indicating that it was independent of diabetes [12]. Taken together, these observations led to our hypothesis that, independent of diabetes, SGLT2 inhibition would cause pro-metabolic programming of the failing heart, thereby leading to improved mitochondrial function and amelioration of aspects of the phenotype that are due to deficient ATP production and/or excessive ROS production.

To test this hypothesis, we studied mice with cardiac-specific Gαq-overexpression, a robust model of non-diabetic heart failure with a reduced ejection fraction (HFrEF). Gαq transduces stimuli, including mechanical strain and neurohormones, that cause pathological remodeling of the heart [13,14]. Transgenic mice with cardiac-specific over-expression of Gαq have proven valuable in understanding the signaling cascades that lead to myocyte hypertrophy and apoptosis [14,15]. These mice develop progressive LV dilation and systolic failure, with hypertrophy and marked apoptosis of cardiac myocytes [15,16]. The hearts of Gαq mice are under increased oxidative stress [16,17] which mediates, in part, many of the cardinal features of the phenotype including myocyte hypertrophy, myocyte apoptosis and interstitial fibrosis - as all are prevented by transgenic overexpression of the antioxidant enzyme catalase in the cytosol [16] or mitochondria [18]. A role for Gαq in determining mitochondrial function is supported by the co-localization of Gαq with mitochondrial membranes [19], and the observation that cardiac myocyte-specific overexpression of Gαq is associated with reprogramming of mitochondrial gene expression and impaired mitochondrial function that contribute to pathologic hypertrophy [17].

## 2 Methods

### 2.1 Experimental animals

Experiments were performed in cardiac-specific Gαq-overexpressing male mice and wild type controls [15], as we have previously described [16]. Body weight and food consumption were measured weekly. Fasting blood glucose was measured in whole blood collected from the tail vein following an overnight fast using a ContourNext glucometer as per the manufacturer's instructions. The protocol was approved by the Institutional Animal Care and Use Committee at Boston University School of Medicine.

### 2.2 Study design

In preliminary experiments, LV myocardium from wild type (WT) and Gαq-overexpressing (Gαq) mice at 4 and 20 weeks of age (*n* = 4 per group) was isolated and subjected to RNA sequence analysis. In the primary experiments, mice at 4 weeks of age were randomized to chow formulated (Research Diets, New Brunswick, NJ) with or without ertugliflozin (ERTU; Merck; 0.5 mg/g of chow), which was continued for 16 weeks, to create four treatment groups each consisting of at least 20 mice as follows: Group 1) WT / control diet (CD); Group 2) Gαq / CD; Group 3) WT / ERTU; and Group 4) Gαq / ERTU. All mice had echocardiograms at 0, 4, 8, 12 and 16 weeks of treatment. From each of the four treatment groups, mice were allocated to one of three types of studies: 1) isolation of mitochondria for measurement of ATP and ROS; 2) tissue for histology and RNA sequencing; or 3) perfused heart studies. There was no overlap of mice used in the three types of studies. The number of mice allocated to each type of study is shown in the Results and Figure legends. Echo data are presented only for those hearts that were successfully used in experiments.

### 2.3 Echocardiography

Two-dimensional and M-mode echocardiography were used to measure cardiac function and dimensions using an Acuson Sequoia C-256 echocardiograph machine equipped with a 15-mHz Model 15L8 transducer, as previously described [16].

### 2.4 Myocardial histology

A piece of LV from each mouse was fixed in 10 % buffered formalin and then embedded in paraffin, sectioned, stained and analyzed, as previously described [16]. In brief, myocyte cross-sectional area was measured in sections stained with hematoxylin and eosin, examined under a light microscope (BX40, Olympus) in 60 myocytes from 10 random fields taken from 2 sections per animal. Apoptosis was assessed using the In Situ Cell Death Detection Fluorescein Kit (Roche Applied Sciences) according to manufacturer's instructions. LV sections were incubated with a reaction mixture containing terminal deoxynucleotidyl transferase and fluorescein-labeled dUTP. Cardiomyocytes were identified by labeling sections with mouse anti-α-sarcomeric actin monoclonal antibody (Sigma-Aldrich) and goat anti-mouse IgG conjugated TRITC (Sigma). Nuclei were identified by Hoechst 33258 stain. Samples were analyzed under a fluorescence microscope (Diaphot 300, Nikon). Cardiomyocyte nuclei were determined by random counting for 10 fields per section in 4 sections per animal. The number of apoptotic nuclei was calculated per 10,000 cardiomyocytes. Fibrosis was assessed by staining sections with the Picrosirius Red Kit (Polysciences). Stained sections were examined under a light microscope (BX40, Olympus). Mean percentage of fibrosis was quantified from 10 random fields taken from 2 sections per animal using NIH ImageJ software. 4-hydroxynonenal (4-HNE) immunohistochemical staining was performed by blocking sections with 10 % goat serum in PBS, then incubating sections with mouse anti-4-HNE monoclonal antibody (Percipio Biosciences), and then incubating with goat biotin-conjugated anti-mouse IgG (Vector Laboratories). Sections were incubated with avidin and biotinylated horseradish peroxidase macromolecular complex (Vector Laboratories) and stained with 3-amino-9-ethylcarbazole (Vector Laboratories) and hematoxylin (Vector Laboratories). Samples were examined under a light microscope (BX40; Olympus) in 2 sections of each heart from 10 color images randomly selected and photographed at a magnification of 40×. Area and intensity were blindly scored for quantification as follows: 0, no visible staining; 1, faint staining; 2, moderate staining; and 3, strong staining.

### 2.5 ATP and H_2_O_2_ production in isolated mitochondria

LV tissue was homogenized and a crude extract of intact mitochondria was prepared with a 2-step centrifugation and mitochondrial H_2_O_2_ production and ATP synthesis were measured, as previously described [20]. In brief, mitochondrial H_2_O_2_ production was measured with Amplex Ultra Red horseradish peroxidase fluorescence method (Invitrogen) using pyruvate (5 mM) and malate (5 mM) as substrates for catalase-inhibited H_2_O_2_ production. H_2_O_2_ production was determined from the slope of the increase in fluorescence (excitation wavelength 545 nm, emission wavelength 590 nm) over a 20-minute period with measurements every 30 s (Tecan M1000 Pro multimode plate reader, room temperature, 10 μg of protein per well).

Maximal ATP synthesis was measured via a luciferin/luciferase-based ATP bioluminescence assay kit (Roche), also using pyruvate and malate as substrates for the oligomycin-sensitive ATP synthesis reaction. ATP production was determined using the initial slope of the increase in ATP-supported luciferase chemiluminescence (~40–60 s, measuring every 6 s) after accounting for background and non-mitochondrial ATP values (Tecan M1000 Pro multimode plate reader, room temperature; 10 μg of protein per well).

### 2.6 LV contractile function and high-energy phosphates in beating hearts

LV contractile function was assessed using a Langendorff heart preparation, as previously described [21]. Briefly, hearts were perfused at a constant pressure of 80 mmHg with Krebs-Henseleit buffer containing glucose (10 mM) and pyruvate (0.5 mM), and coronary flow rates were measured. A water-filled balloon was placed in the LV to measure LV pressure and adjust LV volume. Perfused hearts were placed in a 9.4 T superconducting magnet and maintained at 37 °C throughout the protocol. Hearts paced at 450 bpm were stabilized for 30 min, then balloon volume was adjusted to reach an end diastolic pressure of 8–9 mmHg and held constant during the remainder of the protocol. LV developed pressure was calculated by subtracting diastolic pressure from systolic pressure. LV work demand was changed by increasing the pacing rate from 450 to 600 bpm and by increasing the concentration of CaCl_2_ in the Krebs-Henseleit buffer from 2 to 4 mM. Work performed was calculated by multiplying the developed pressure by heart rate. High energy phosphates (ATP, phosphocreatine and inorganic phosphate) were measured simultaneously using ^31^P nuclear magnetic resonance spectroscopy, as we have previously described [22].

### 2.7 RNA isolation and sequencing

RNA was isolated from frozen LV, sequenced and analyzed as previously described [12]. In brief, LV from 4 to 5 hearts per treatment group was homogenized, and mRNA extracted with the RNeasy Universal Mini kit (Qiagen) according to the manufacturer's instructions. The Boston University Microarray and Sequencing Resource Core Facility performed RNA quantity and quality measurements, library preparation, and RNA sequencing. RNA integrity was confirmed via RNA Pico assay for total RNA Bioanalyzer QC analysis (Agilent). Sequencing libraries were generated using the NEBNext Ultra II RNA Kit with poly(A) selection and run on an Illumina NextSeq 2000 platform with 100-bp paired-end reads. FASTQ files were aligned to mouse genome build mm10 using STAR (version 2.6.0c) (Dobin et al., 2013). Ensembl-Gene-level counts for nuclear-encoded genes were generated using featureCounts (Subread package, version 1.6.2) and Ensembl annotation build 100 (uniquely aligned proper pairs, same strand). FASTQ quality was assessed using FastQC (version 0.11.7), and alignment quality was assessed using RSeQC (version 3.0.0). Variance-stabilizing transformation (VST) was accomplished using the varianceStabilizingTransformation function in the DESeq2 R package (version 1.32.0) (Love et al., 2014). Differential expression was assessed using the Wald test implemented in the DESeq2 R package. Correction for multiple hypothesis testing was accomplished in DESeq2 using the Benjamini-Hochberg false discovery rate (FDR) after excluding genes with low overall expression using the “independent filtering” step (the default behavior in DESeq2). All analyses were performed using the R environment for statistical computing (version 4.1.2).

### 2.8 GeneSet enrichment analysis

Gene Set Enrichment Analysis (GSEA; version 2.2.1) [23] was used to identify biological terms, pathways and processes that are coordinately up- or down-regulated with respect to experimental variables, as we have described [12]. In brief, the Entrez Gene identifiers of all genes in the Ensembl Gene annotation were ranked by the Wald statistic computed for each effect in each two-factor model and for each pairwise comparison. Ensembl Genes matching multiple mouse Entrez Gene identifiers were excluded prior to ranking, so that the ranked list represents only those Entrez Gene IDs that match exactly one Ensembl Gene. Each ranked list was then used to perform pre-ranked GSEA analyses (default parameters with random seed 1234) using the Entrez Gene versions of the mouse Hallmark (MH) and Gene Ontology (GO) (M5) gene sets collections obtained from the Molecular Signatures Database (MSigDB), version 2024.1.Mm [23].

### 2.9 KEGG pathway visualization

When DESeq2 was used to perform Wald tests with each two-factor model, log2 fold change values were also computed for each effect in each model. These log2 fold change values were used as input to KEGG (https://www.genome.jp/kegg/) as follows: first, the values for any genes that were not well-expressed (i.e., did not pass the independent filtering step) were excluded; then, the remaining log2 fold change values were averaged together for each object (i.e., gene product or EC number) in a given KEGG pathway; finally, the mean log2 fold change values were converted to color gradations using the KEGG Mapper Color tool in Min-Mid-Max mode, assigning blue, white and red to values of ≤−1, 0 and ≥+1, respectively. Objects that were not associated with any genes (or were associated with mitochondrially encoded genes) were excluded from visualization.

### 2.10 Data availability

The data discussed have been deposited in NCBI's GEO [24] and are accessible through GEO Series accession number GSE276290 (https://www.ncbi.nlm.nih.gov/geo/query/acc.cgi?acc=GSE276290).

### 2.11 Statistical analysis

All data are presented as mean ± SEM. Significance was determined using a 1-way ANOVA with Bonferroni correction for multiple comparisons. GraphPad Prism 9 software was for used statistical analysis. A *p* < 0.05 was considered significant.

## 3 Results

### 3.1 Body weight and fasting glucose

In wild type mice ERTU treatment mildly decreased body weight at 20 weeks of age (after 16 weeks of treatment) and caused a similar non-significant trend in Gαq mice (Suppl. Fig. 1A). ERTU treatment had no effect on fasting blood glucose levels at 20 weeks of age in either genotype (Suppl. Fig. 1B).

### 3.2 ERTU treatment arrests Gαq-induced structural remodeling and contractile dysfunction (Fig. 1A-E; and Suppl. Fig. 2)

Gαq mice developed pathologic cardiac remodeling with LV dilation, thinning and contractile dysfunction. At 4 weeks of age, prior to initiation of ERTU treatment, heart rate was decreased, LV end-diastolic dimension (EDD) and end-systolic dimension (ESD) were increased, and wall thickness and fractional shortening were decreased in Gαq animals compared to wild type littermate controls (*p* < 0.001 for all). Over the ensuing 16 weeks, Gαq hearts continued to dilate and fail further, as evidenced by increases in EDD and ESD, and a further decrease in fractional shortening. ERTU treatment, which was started at 4 weeks of age, arrested LV dilation, thinning and contractile failure in Gαq animals, but had no effect on heart rate.

**Fig. 1:**
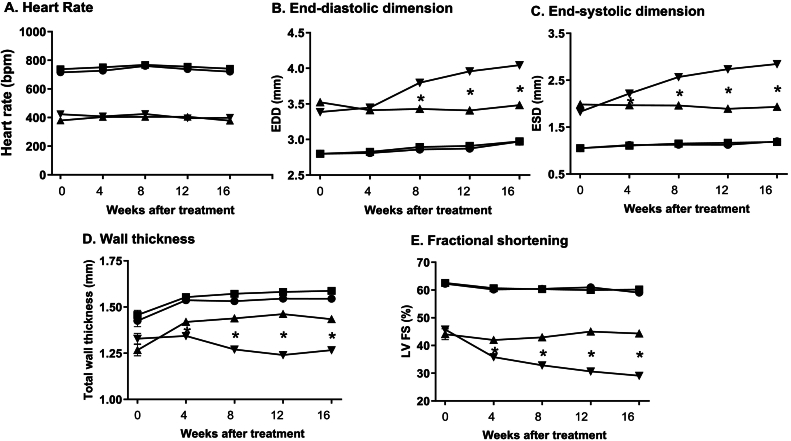
ERTU prevents left ventricular (LV) dilation, wall thinning and systolic failure in Gαq mice. Beginning at 4 weeks of age (0 weeks after treatment in all panels), wild type (WT) and cardiac-specific Gαq-overexpressing mice were randomized to chow formulated with ertugliflozin (ERTU; 0.5 mg/g of chow) or control diet without ERTU (CD). Over the subsequent 16 weeks, Gαq mice exhibited decreased heart rate (Panel A), progressive LV dilation (Panels B and C) with LV wall thinning (Panel D) and a decrease in LV fractional shortening (Panel E) as assessed by echocardiography. Treatment with ERTU prevented LV dilation, wall thinning and progressive systolic dysfunction. Data presented as mean ± SEM; *n* = 10–16 mice/group); * = *p* < 0.001 Gαq vs. Gαq ERTU. ■ = WT / CD, ● = WT / ERTU, ▼ = Gαq / CD, ▲ = Gαq / ERTU. Individual mouse data at 16 weeks of treatment are shown in Suppl. Fig. 2.

### 3.3 ERTU treatment prevents the development of apoptosis, myocyte hypertrophy and fibrosis in Gαq mice

**Fig. 2:**
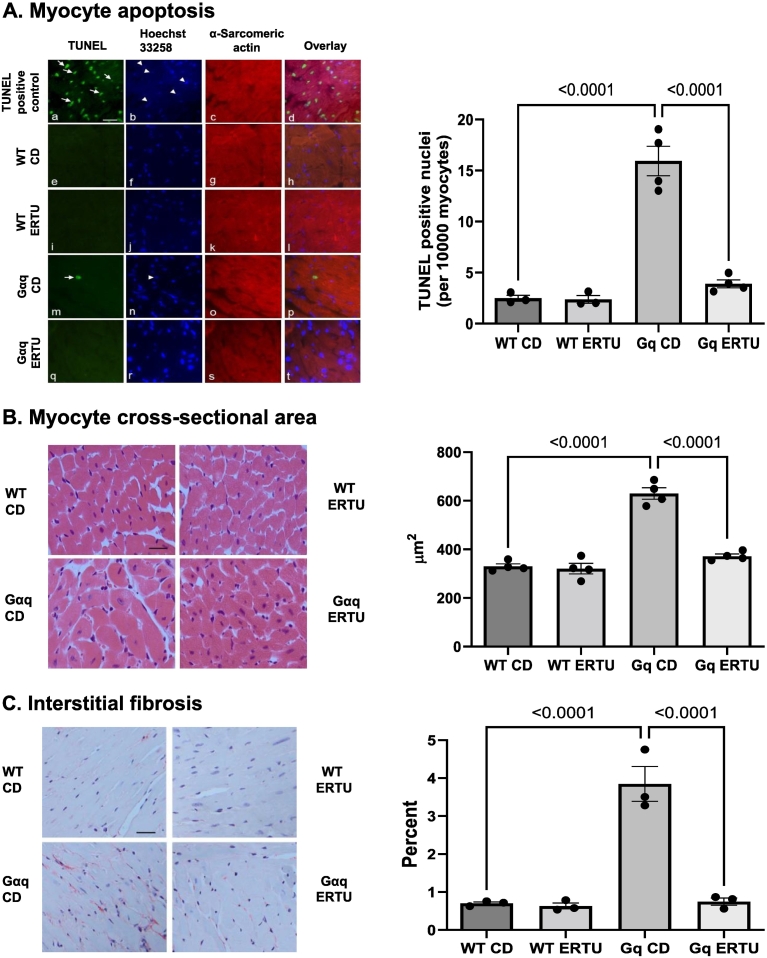
ERTU prevents myocyte apoptosis and cellular remodeling. WT and Gαq mice were sacrificed for histologic analysis after 16 weeks of treatment. Gαq mice exhibited marked myocyte apoptosis, myocyte hypertrophy and interstitial fibrosis, all of which were prevented in ERTU-treated hearts. Panel 2A. Myocyte apoptosis was assessed by TUNEL staining: Apoptotic nuclei (arrows) are shown by green fluorescence in Panels a, e, i, m and q. Nuclei (arrowheads) stained by Hoechst 33258 are shown in blue in Panels b, f, j, n and r. Cardiomyocytes identified by the overlays in Panels d, h, l, p and t allow identification of apoptotic nuclei present in myocytes. Panel 2B. Myocyte hypertrophy was assessed by measurement of cross-sectional areas. Panel 2C. Interstitial fibrosis was assessed by picrosirius red staining. Data are presented as mean ± SEM; *n* = 3–4 mice/group. (For interpretation of the references to color in this figure legend, the reader is referred to the web version of this article.)

Apoptosis, a cardinal feature in the Gαq heart, was markedly increased in Gαq control hearts as assessed by TUNEL staining, and the increase was prevented by ERTU treatment (Fig. 2A). Hypertrophy of cardiac myocytes, another cardinal feature of the Gαq heart, was present in Gαq control hearts, and was prevented at 16 weeks of ERTU treatment (Fig. 2B). Likewise, interstitial fibrosis, measured by picrosirius red staining, was increased in Gαq control hearts, and the increase was prevented by ERTU treatment (Fig. 2C).

### 3.4 ERTU treatment prevents mitochondrial dysfunction and oxidative stress in Gαq mice

**Fig. 3:**
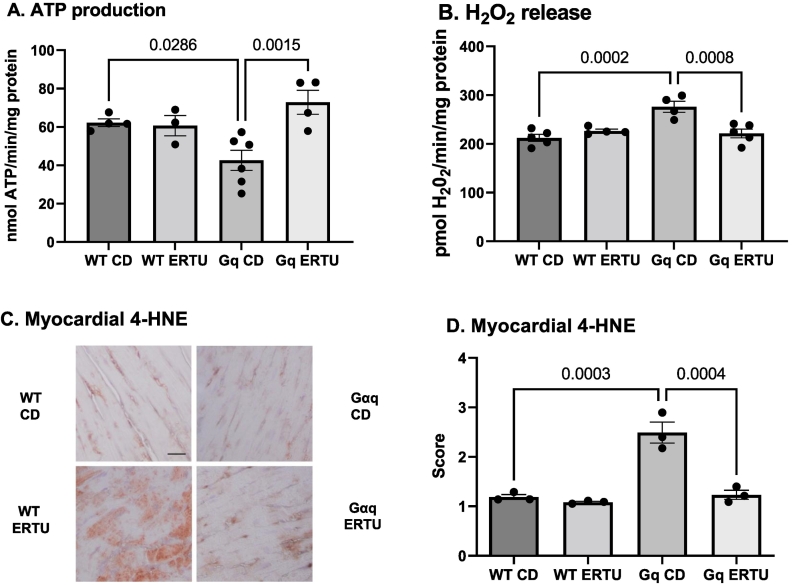
ERTU prevents mitochondrial dysfunction and myocardial oxidative stress in Gαq mice. In cardiac mitochondria isolated from LV of Gαq mice after 16 weeks of treatment, ATP production was decreased (Panel A) and H_2_O_2_ release was increased (Panel B). Both ATP production and H_2_O_2_ release were normalized by ERTU treatment. Consistent with the increased H_2_O_2_ release, myocardial oxidative stress, as assessed by immunohistochemical staining for 4-hydroxynonenal (4-HNE), was increased in Gαq mice, and the increase was prevented by ERTU treatment. Shown are representative images of 4-HNE staining (Panel C; scale bar = 25 μm), and the mean scoring for 4-HNE staining (Panel D). Data presented are mean ± SEM; *n* = 4–6 mice/group for Panels A and B, and 3 mice/group for Panel D.

Mitochondrial function was assessed in myocardial mitochondria isolated after 16 weeks of treatment. Maximal ATP production was decreased in Gαq mitochondria, and the decrease was prevented by ERTU treatment (Fig. 3A). Conversely, H_2_O_2_ release was increased in myocardial mitochondria isolated from Gαq mice and was prevented by ERTU treatment (Fig. 3B). In parallel studies, the myocardial level of 4-HNE, a marker of tissue oxidative stress, was markedly increased in Gαq hearts and the increase was prevented by ERTU treatment (Fig. 3C, D).

### 3.5 ERTU treatment improves contractile reserve and energetics in Gαq hearts

**Fig. 4:**
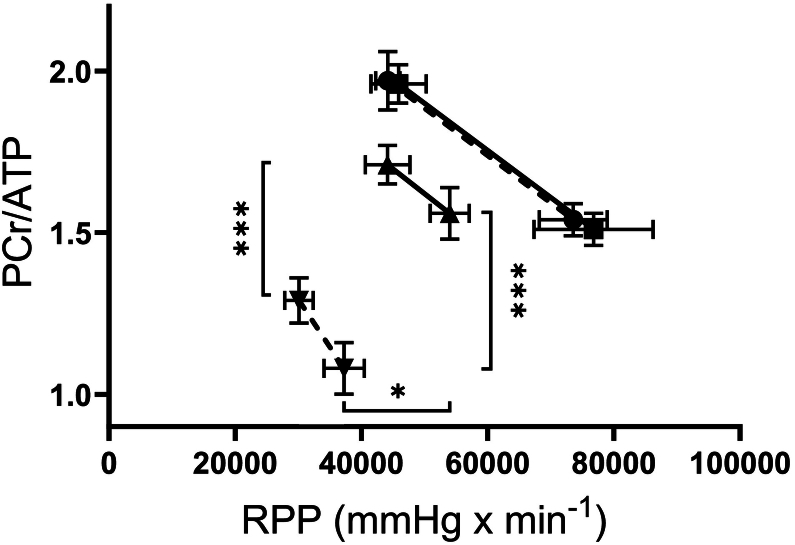
Effect of ERTU on myocardial high energy phosphates and cardiac contractile reserve. After 16 weeks of treatment, phosphocreatine (PCr) and ATP were measured by ^31^P NMR simultaneously with contractile function in isolated beating hearts while increasing work demand by raising the pacing rate and the concentration of calcium in the perfusate. Contractile reserve was assessed as the change in rate X pressure product (RPP) with increased work demand. In hearts from Gαq mice (▼, dashed line) RPP failed to increase with increased work demand and was associated with depressed levels of PCr/ATP both with basal and increased work demand. In hearts from Gαq mice, ERTU treatment (▲, solid line) increased RPP and PCr/ATP at all levels of work demand thereby shifting the relationship between RPP and PCr/ATP upward and to the right, indicative of improved energy balance at higher levels of contractile performance. In wild type mice (■, solid line), ERTU (●, dashed line) had little effect. *** = 0.001; ** = 0.01, * = 0.05; data presented as mean ± SEM; *n* = 5–7 mice/group). See also Table 1.

Myocardial contractile reserve and high energy phosphates were measured in beating hearts at baseline and with increased work demand. Hearts from Gαq mice had decreased contractile reserve, as reflected by the RPP, both at baseline and at high work demand (Fig. 4 and Table 1). ERTU treatment prevented the decrease in RPP at baseline and partially prevented the decrease at high work demand.

**Table 1:** Effect of ERTU on isolated beating heart function and myocardial energetics.

	WT (*n* = 7)		WT ERTU (n = 5)		Gαq (n = 7)		Gαq ERTU (*n* = 6)	
	BL	Peak	BL	Peak	BL	Peak	BL	Peak
End-diastolic pressure (mmHg)	8.9	12.4	8.8	12.3	9.9	26.4	9.0	15.7
	± 0.1	± 1.3	± 0.3	± 1.5	± 0.5	± 6.4^⁎⁎^	± 0.2	± 1.3^#^
End-systolic pressure (mmHg)	111.6	154.7	110.8	153.8	76.8	88.6	107.2	105.7
	± 8.3	± 24.2	± 9.6	± 27.1	± 4.6	± 5.9^⁎⁎^	± 7.8	± 5.7
Developed pressure (mmHg)	98.7	122.7	102.0	128.0	66.9	62.1	98.2	90.0
	± 4.2	± 9.0	± 9.7	± 15.5	± 4.9^⁎^	± 5.3^⁎⁎⁎^	± 7.9^#^	± 4.9^#^
RPP (10^3^ mmHg/min)	44.2	73.6	45.9	76.8	30.1	37.3	44.2	54.0
	± 1.9	± 5.4	± 0.7	± 0.45	± 2.2	± 3.2^⁎⁎⁎^	± 3.5	± 2.9^#^
Coronary flow (ml/min)	2.49 ± 0.10	3.01 ± 0.10	2.60 ± 0.13	3.19 ± 0.15	3.17 ± 0.16^⁎⁎^	3.77 ± 0.13^⁎⁎⁎^	3.43 ± 0.16^⁎⁎^	3.95 ± 0.14^⁎⁎^
PCr/ATP	1.97 ± 0.09	1.54 ± 0.05	1.95 ± 0.05	1.51 ± 0.05	1.29 ± 0.06^⁎⁎⁎^	1.08 ± 0.07^⁎⁎⁎^	1.71 ± 0.05^###^	1.54 ± 0.08^###^

Contractile function and high energy phosphates (^31^P NMR spectroscopy) were measured simultaneously in isolated retrograde-perfused Langendorff hearts. At baseline (BL) hearts were paced at 450 beats per minute and perfused with 2 mM CaCl_2_. At high workload (Peak) hearts were paced at 600 beats per minute and perfused with 4 mM CaCl_2_. Data are presented as mean ± SEM (* p < 0.05, ** p < 0.01 and *** p < 0.001 vs. WT at corresponding workload; # *p* < 0.05, ## *p* < 0.01and ### *p* < 0.001 vs. Gαq at corresponding workload; n = 5–7 mice per condition as noted.

PCr and the PCr/ATP ratio were decreased in Gαq control hearts compared to wild type hearts, both at baseline and with high work demand (Fig. 4 and Table 1). In Gαq hearts, ERTU treatment partially restored the PCr and the PCr/ATP ratio at baseline and prevented the decrease with high work demand. The relationship between PCr/ATP and RPP, which reflects overall energetic performance, was markedly depressed in Gαq hearts, and was shifted upward and rightward by ERTU, indicating improved energy balance at higher levels of contractile performance. In wild type hearts, ERTU treatment had no effect on the relationship between PCr/ATP and RPP.

### 3.6 Depressed expression of metabolic genes in Gαq mice is partially corrected by ERTU

**Fig. 5:**
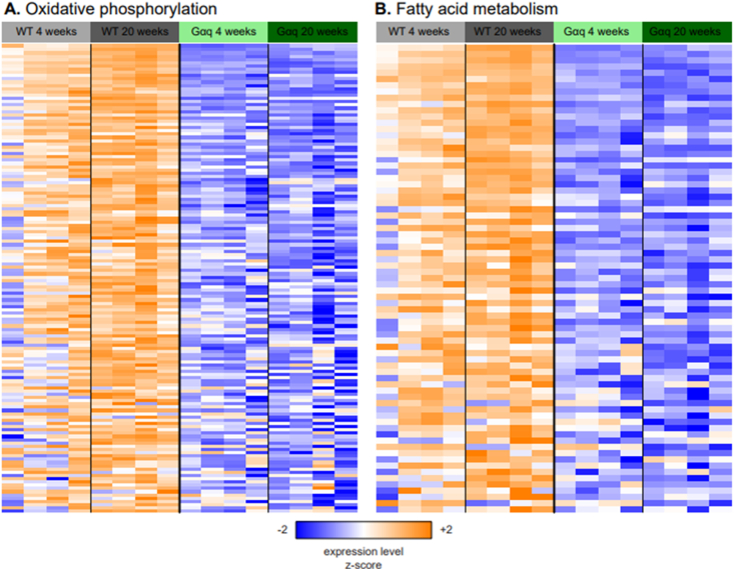
Gαq over-expression suppresses myocardial gene sets associated with mitochondrial function at 4 and 20 weeks of age. Myocardial gene expression was assessed by mRNA sequencing with gene set enrichment analysis (GSEA). Of the most suppressed Gene Ontology (GO) gene sets, most were related to mitochondrial structure and/or function (see also Suppl. File 1 for full gene set listing and statistics). Further analysis by Hallmark GSEA (shown here) indicated that the gene sets for oxidative phosphorylation (OXPHOS; Panel A; NES -3.26, *p* < 0.001, q < 0.001) and fatty acid oxidation (FAO; Panel B; NES -2.83, p < 0.001, q < 0.001) were markedly suppressed at both 4 and 20 weeks of age in Gαq mice. Variance-stabilizing-transformed gene expression values were z-score-normalized (to a mean of zero and standard deviation of one) within each row with blue, white and orange indicating z-scores of ≤−2, 0, and ≥+2, respectively (*n* = 4 mice/group). (For interpretation of the references to color in this figure legend, the reader is referred to the web version of this article.)

In preliminary studies, mRNA isolated from the hearts of wild type and Gαq mice at 4 and 20 weeks of age was subjected to RNA sequencing, and the significance of each gene was determined with respect to each effect in a model of expression as a function of genotype and timepoint, or the comparison between Gαq and wild type at each timepoint. Gene Set Enrichment Analysis (GSEA) was then used to determine which gene sets from the Gene Ontology (GO) and Hallmark collections of the Molecular Signatures Database (MSigDB, v2024.1.Mm) demonstrated significant coordinate up- or down-regulation with respect to each model effect or pairwise comparison. This analysis identified 294 gene sets that were significantly coordinately up- or down-regulated with respect to genotype after correcting for timepoint (FDR *q* < 0.05) (Supplementary File 1); of these, 186 were also significant (FDR *q* < 0.05) in the same direction for the comparison between Gαq and wild-type at 4 weeks, and 260 were significant at 20 weeks. The gene sets with the strongest coordinate downregulation by Gαq were related to mitochondrial structure and/or function, including cellular respiration, oxidative phosphorylation, electron transport and fatty acid oxidation. The expression of the most strongly downregulated (leading edge) genes from two of these pathways, the oxidative phosphorylation and fatty acid metabolism gene sets of the Hallmark collection, is shown in these samples in Fig. 5.

**Fig. 6:**
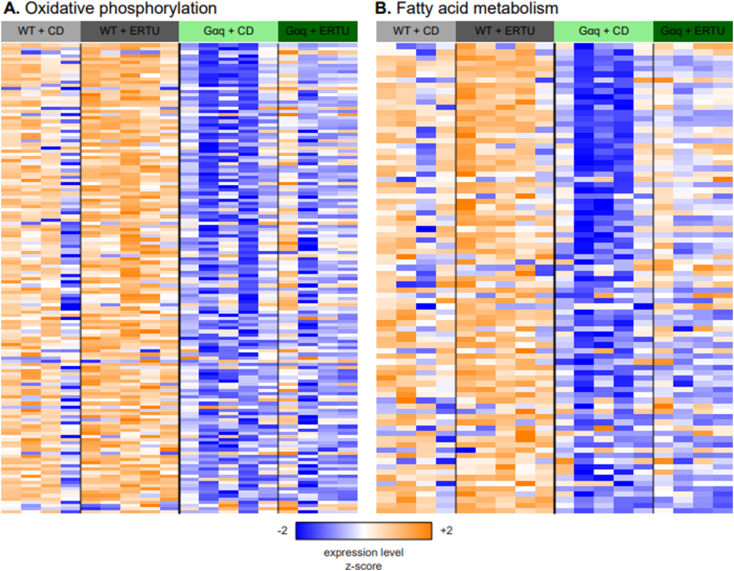
ERTU treatment up-regulates myocardial genes for oxidative phosphorylation and fatty acid metabolism. After 16 weeks of treatment with ERTU, the most enriched GO gene sets were for mitochondrial structure and function (see also Suppl. File 2 for full gene set listing and statistics). Hallmark GSEA showed that ERTU treatment markedly enriched gene sets for oxidative phosphorylation (OXPHOS; Panel A; NES 2.97, *p* < 0.0001, q < 0.0001) and fatty acid metabolism (FAO; Panel B; NES 2.61, p < 0.0001, q < 0.0001). Variance-stabilizing-transformed gene expression values were z-score-normalized (to a mean of zero and standard deviation of one) within each row with blue, white and orange indicating z-scores of ≤−2, 0, and ≥+2, respectively (n = 4–5 mice/group). (For interpretation of the references to color in this figure legend, the reader is referred to the web version of this article.)

In the same manner, mRNA from the hearts of wild type and Gαq mice fed either a control diet (CD) or an ERTU diet for 16 weeks was isolated and sequenced, and the significance of each gene was determined with respect to each effect in a model of expression as a function of genotype and diet, or the comparison between ERTU and CD in each genotype. GSEA identified 426 gene sets that were significantly coordinately up- or down-regulated with respect to diet after correcting for genotype (FDR *q* < 0.05) (Supplementary File 2); of these, 132 were also significant (FDR *q* < 0.05) in the same direction for the comparison between ERTU and CD in WT animals, and 107 in Gαq animals. The gene sets with the most significant coordinate upregulation by ERTU were largely the same as those that were downregulated by Gαq and were related to aerobic respiration and fatty acid oxidation. The expression of the leading-edge genes from the oxidative phosphorylation and fatty acid metabolism gene sets of the Hallmark collection is shown in these samples in Fig. 6. GSEA performed for ERTU vs. CD in only the wild-type mice showed significant coordinate up-regulation for gene sets for both oxidative phosphorylation (NES 3.21, *p* < 0.001, q < 0.001) and fatty acid metabolism (NES 2.44, p < 0.001, q < 0.001) (Supplementary File 2).

**Fig. 7:**
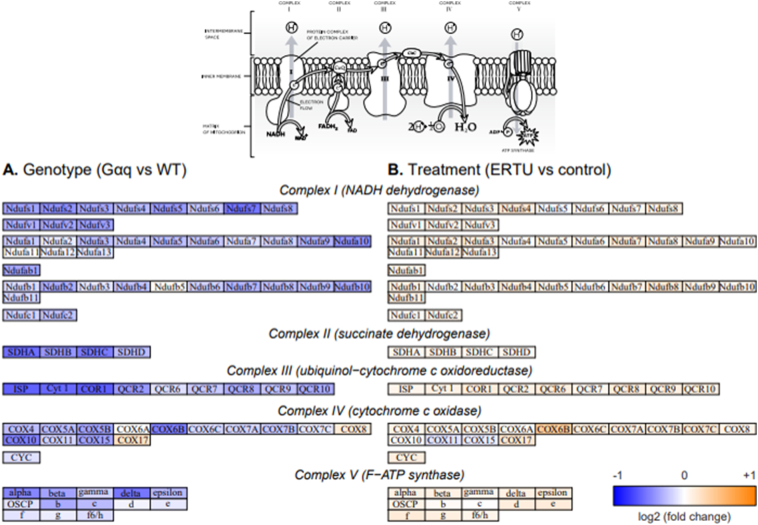
Kegg pathway depiction of mitochondrial genes for oxidative phosphorylation arranged by mitochondrial complex. Panel A depicts the change in gene expression for Gαq vs. wild type mice at 20 weeks of age. Panel B depicts the changes in gene expression for mice at 20 weeks of age treated with ERTU vs. control diet for 16 weeks. Blue, white and orange indicate log2 fold change values of ≤−1, 0 and ≥+1, respectively. (For interpretation of the references to color in this figure legend, the reader is referred to the web version of this article.)

**Fig. 8:**
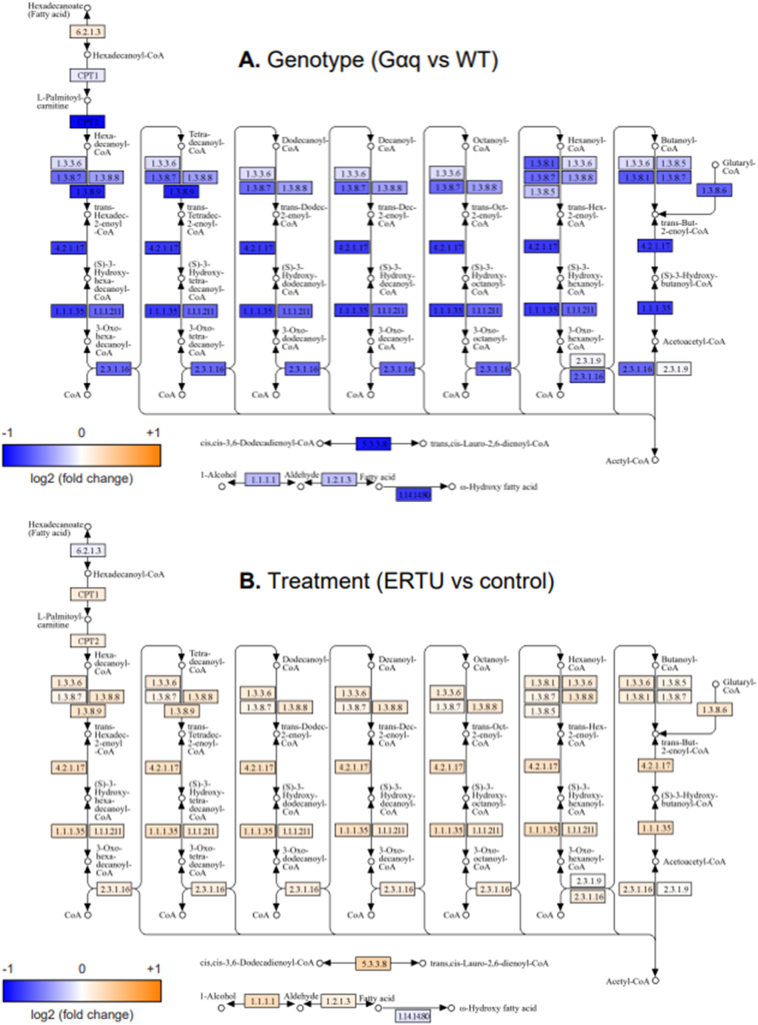
A. Kegg pathway depicting mitochondrial genes for fatty acid degradation for Gαq vs. wild type mice at 20 weeks of age. Blue, white and orange indicate log2 fold change values of ≤−1, 0 and ≥+1, respectively. B. Kegg pathway depicting mitochondrial genes for fatty acid degradation for Gαq vs. wild type mice at 20 weeks of age treated with ERTU vs. control diet for 16 weeks. Blue, white and orange indicate log2 fold change values of ≤−1, 0 and ≥+1, respectively. (For interpretation of the references to color in this figure legend, the reader is referred to the web version of this article.)

As a more granular way to visualize these changes in gene expression, the log2 fold changes that were computed for each effect in the model of expression as a function of genotype and diet were used to shade the components of the murine KEGG pathways “oxidative phosphorylation” (Fig. 7) and “fatty acid degradation” (Fig. 8). This confirmed that most genes involved in both pathways were downregulated in Gαq mice (vs. wild type), and conversely, that treatment with ERTU (vs. CD) increased gene expression in both pathways.

## 4 Discussion

### 4.1 Mitochondrial dysfunction in heart failure

Mitochondrial dysfunction is a central feature of the failing heart leading to depressed production of ATP and excessive release of ROS [1]. We used an SGLT2 inhibitor, ERTU, to counteract metabolic aberrations associated with heart failure and to elucidate the role of mitochondrial dysfunction in the progression of dilated cardiomyopathy in a mouse model with cardiac myocyte-specific overexpression of Gαq. There are three major new findings. First, excessive Gαq signaling caused maladaptive reprogramming of mitochondrial genes at 4 weeks of age. Second, treatment of Gαq mice with the SGLT2 inhibitor led to substantial correction of mitochondrial gene expression that was associated with a) normalization of mitochondrial ATP production, b) restoration of the energetic response to work demand, c) elimination of excessive mitochondrial ROS release, and d) correction of myocardial oxidative stress. Third, ERTU prevented Gαq-stimulated cellular remodeling events (myocyte hypertrophy, myocyte apoptosis, and interstitial fibrosis) and prevented the progression of adverse structural remodeling and contractile failure. Taken together, these observations support the thesis that Gαq-stimulated mitochondrial dysfunction contributes to adverse energetic and structural remodeling.

### 4.2 Gαq mediates adverse myocardial remodeling

Gαq transduces stimuli for many of the signaling cascades that are involved in pathological remodeling with LV hypertrophy, dilation and contractile failure [13,15,16]. Hypertrophy and apoptosis of cardiac myocytes and interstitial fibrosis are prominent features of the Gαq mouse heart that recapitulate the cellular events in dilated cardiomyopathy. Of note, Westenbrink and colleagues showed that mitochondrial dysfunction is an important feature of the Gαq mouse heart that is associated with apoptosis, impaired mitochondrial respiration and abnormalities in mitochondrial gene expression that are mediated, in part, by CaMKIIδ [17]. Gαq proteins localize with mitochondrial membranes where they appear to participate in the regulation of mitochondrial biology and respiratory function via non-canonical Gαq signaling and crosstalk between Gαq and mitochondria [24].

### 4.3 SGLT2 inhibition upregulates metabolic gene expression and function

We previously observed that chronic ERTU administration restored expression of myocardial gene sets for oxidative phosphorylation and fatty acid metabolism; and corrected mitochondrial dysfunction in mice with diet-induced diabetic cardiomyopathy [12]. Notably, the restoration of these pro-metabolic gene sets was not confined to the diabetic state, as SGLT2 inhibition also induced similar effects in non-diabetic control mice. Likewise, in the present study in non-diabetic Gαq mice, ERTU treatment, beginning at 4 weeks of age, significantly reprogrammed mitochondrial gene expression towards baseline levels and fully restored isolated mitochondrial function by 20 weeks of age, as evidenced by normalization of ATP production and ROS release. Our observations are consistent with the recent suggestion that SGLT2 inhibitors stimulate PANK1, a rate-limiting regulator of coenzyme A (Co-A) synthesis, thereby correcting a deficiency in Co-A and improving fatty acid utilization [25]. In this context, our findings suggest that mitochondrial gene regulation may depend on the mitochondrial capacity to metabolize substrates which can be constrained in the failing heart due to Co-A deficiency. ERTU also upregulated genes for oxidative phosphorylation and fatty acid metabolism in wild type mice, but in contrast to the effects in Gαq mice, did not enhance mitochondrial function or energetics beyond normal levels. This suggests that under these experimental conditions, upregulating mitochondrial oxidative capacity provides no additional functional benefit in healthy hearts. This study adds to the growing evidence of therapeutic potential of SGLT2 inhibitors by demonstrating their ability to enhance cardiac energetics in failing hearts even in the absence of diabetes. In non-diabetic pigs [7] and mice subjected to myocardial infarction [26], SGLT2 inhibition ameliorated adverse remodeling and led to normalization of glucose and fatty acid metabolism. Likewise, in mice with pressure overload induced by aortic constriction, the SGLT2 inhibitor empagliflozin improved oxidative phosphorylation, enhanced autophagy and decreased cardiac apoptosis [9]. Our study not only confirms the beneficial effect of SGLT2 inhibition on metabolic function in the non-diabetic heart, but also links this effect to reprogramming of metabolic genes that regulate mitochondrial structure and function. The functional importance of metabolic reprogramming is further supported by our demonstration that ERTU improved energetic and contractile function ex vivo in beating hearts from Gαq mice with dilated cardiomyopathy. Thus, our findings not only confirm the metabolic benefits of SGLT2 inhibition, but also establish a novel link to the regulation of mitochondrial structure and function in the non-diabetic failing heart.

### 4.4 ERTU prevented progression of adverse myocardial remodeling

Over the 16-week treatment course, ERTU arrested the progression of left ventricular (LV) dilation and functional decline in Gαq mice. Likewise, myocyte hypertrophy, myocyte apoptosis and interstitial fibrosis were absent after ERTU treatment for 16 weeks. Despite this, the initial LV dilation and contractile impairment that was present prior to treatment at 4 weeks persisted, despite ERTU treatment. Likewise, in Langendorff heart studies performed at 20 weeks of age, ERTU treatment led to marked, albeit incomplete, recovery of contractile function as reflected by the RPP. The most likely explanation for the incomplete contractile recovery is that restored mitochondrial function was not sufficient to compensate for irreversible apoptotic myocyte loss that had occurred by 4 weeks of age. Although cardiac dilation, wall thinning and augmented wall stress likely contributed to increased energy demand, ERTU corrected myocardial energy balance, suggesting that restored oxidative phosphorylation provided adequate ATP to satisfy the elevated energetic needs of the dilated heart.

### 4.5 SGLT2 inhibition suppressed myocardial ROS

A hallmark of mitochondrial dysfunction is the release of excessive ROS that plays a central role in the pathophysiology of the Gαq mouse [[16], [17], [18]]. SGLT2 inhibition normalized ROS production in mitochondria isolated from the Gαq heart and ameliorated oxidative stress present in the myocardium, as reflected by the level of 4-HNE, a marker of myocardial lipid oxidation. Thus, an important consequence of improved mitochondrial function with SGLT2 inhibition is suppression of excessive mitochondrial ROS release that drives cellular processes that underlie pathologic remodeling. This thesis is supported by the similar beneficial effect of transgenic catalase on remodeling in the Gαq mouse [16,18].

### 4.6 Limitations

A limitation of this study is the absence of fatty acids from the substrates for isolated mitochondria and perfused heart experiments. Since fatty acids are a crucial energy source for the healthy heart, this may have contributed, at least in part, to the observed energetic deficit in Gαq hearts. However, given that hypertrophied and failing hearts tend to rely more on glycolysis for energy production the buffer composition likely favored Gαq hearts [27]. Further, since ERTU upregulated genes for fatty acid oxidation, the lack of fatty acids in the perfusion buffer may have underestimated the full effect of ERTU. Another limitation is the use of ERTU, rather than one of the FDA-approved SGLT2 inhibitors for heart failure treatment. While ERTU shares on-target pharmacologic effects with other SGLT2 inhibitors, we cannot confirm that the observed benefits would extend to FDA-approved agents. The dose used in this study elicited maximal glucose excretion in rats, ensuring effective SGLT2 inhibition [28]. However, the serum concentration achieved with this dose in mice may have exceeded clinical concentrations in humans, and ERTU may have acted on additional off-target (i.e., non-SGLT2) pathways [29]. Finally, while we show that the ERTU-mediated improvement in mitochondrial function is associated with improvements in structural and functional remodeling, we cannot exclude the possibility that the effects of ERTU on metabolic function are secondary to improved cardiac function rather than a direct effect on mitochondria.

### 4.7 Summary

This study demonstrates that the SGLT2 inhibitor ERTU can reverse the downregulation of myocardial genes involved in oxidative phosphorylation and fatty acid metabolism observed at 4 weeks in Gαq overexpressing mice. Restoration of these gene programs is associated with correction of mitochondrial dysfunction, marked improvement in myocardial energetics and prevention of ROS-mediated features of myocardial remodeling. Collectively, our observations suggest that an important mechanism of the beneficial effects of SGLT2 inhibitors is related to restoration of mitochondria-related gene networks essential for cardiac function.

The following are the supplementary data related to this article.

Supplementary figures Image 1

Supplementary File 1 GSEA results for experiment involving genotype (Gαq and wild-type, WT) at 4 and 20 weeks. Gene Set Enrichment Analysis (GSEA) was performed using lists of genes ranked by Wald statistics computed from a model of expression as a function of genotype and timepoint, or from Gαq vs WT within each timepoint. This Excel file includes one row for each of the gene sets from the MH (Hallmark) and Gene Ontology (M5) collections of MSigDB v2024.1.Mm. Columns A-C include the group, name, and size of each gene set, and columns D-O include the following information for each ranked list: Normalized Enrichment Score (NES, the ratio of the gene set's Enrichment Score (ES) to the average ES of 1000 randomly selected gene sets of the same size); the nominal *p* value; and the FDR-adjusted p value (q value). Nominal p and FDR q values of zero indicate those gene sets in which none of the 1000 random permutations produced an ES that was higher than the ES computed for the gene set. FDR q values <0.25 are in bold italic font and shaded in a graded fashion such that white and dark orange indicate FDR q values of 0.25 and 0, respectively. Supplementary File 1

Supplementary File 2 GSEA results for experiment involving 16 weeks of ertugliflozin (ERTU) treatment of both Gαq and WT mice. Gene Set Enrichment Analysis (GSEA) was performed using lists of genes ranked by Wald statistics computed from a model of expression as a function of genotype and diet, or from ERTU vs control diet (CD) within each genotype. This Excel file includes one row for each of the gene sets from the MH (Hallmark) and Gene Ontology (M5) collections of MSigDB v2024.1.Mm. Columns A-C include the group, name, and size of each gene set, and columns D-O include the following information for each ranked list: Normalized Enrichment Score (NES, the ratio of the gene set's Enrichment Score (ES) to the average ES of 1000 randomly selected gene sets of the same size); the nominal *p* value; and the FDR-adjusted p value (q value). Nominal p and FDR q values of zero indicate those gene sets in which none of the 1000 random permutations produced an ES that was higher than the ES computed for the gene set. FDR q values <0.25 are in bold italic font and shaded in a graded fashion such that white and dark orange indicate FDR q values of 0.25 and 0, respectively. Supplementary File 2

## CRediT authorship contribution statement

**Jordan M. Chambers:** Writing – original draft, Investigation, Formal analysis. **Dominique Croteau:** Writing – review & editing, Investigation, Formal analysis. **David R. Pimentel:** Writing – review & editing, Supervision, Methodology, Investigation. **Adam C. Gower:** Writing – original draft, Investigation, Formal analysis. **Marcello Panagia:** Writing – review & editing, Supervision, Methodology. **Tomas Baka:** Writing – review & editing, Investigation. **Fuzhong Qin:** Writing – review & editing, Supervision, Methodology, Investigation. **Ivan Luptak:** Writing – review & editing, Supervision, Methodology, Investigation. **Wilson S. Colucci:** Writing – review & editing, Writing – original draft, Supervision, Funding acquisition, Conceptualization.

## Declaration of Generative AI and AI-assisted technologies in the writing process

The authors did not use generative AI or AI-assisted technologies in the development of this manuscript.

## Sources of funding

Supported in part by NIH grants HL-064750 (WSC), HL-166606 (IL), and K08-HL-123744 (MP); a Fellow-to-Faculty Award from the 10.13039/100000968American Heart Association 15FTF25890062 (IL); and an investigator-initiated research grant from the Investigators Studies Research Program of Merck Sharp & Dohme Corp in collaboration with 10.13039/100004319Pfizer. RNA sequencing analysis (ACG) was supported by the BU Microarray and Sequencing Resource and Clinical and Translational Science Award UL1-TR001430. The opinions expressed in this paper are those of the investigators and do not necessarily represent those Merck Sharp & Dohme Corp or Pfizer.

## Declaration of competing interest

The authors declare the following financial interests/personal relationships which may be considered as potential competing interests: Wilson S Colucci reports financial support was provided by National Institutes of Health. Marcello Panagia reports financial support was provided by National Institutes of Health. Ivan Luptak reports financial support was provided by American Heart Association Inc. Wilson S Colucci reports financial support was provided by Merck & Co Inc. If there are other authors, they declare that they have no known competing financial interests or personal relationships that could have appeared to influence the work reported in this paper.

Supported by an investigator-initiated research grant from the Investigators Studies Research Program of Merck Sharp & Dohme Corp in collaboration with Pfizer.
