# Supplementary material for: SGLT2 inhibitor upregulates myocardial genes for oxidative phosphorylation and fatty acid metabolism in Gαq-mice
Source: J Mol Cell Cardiol Plus. 2025 Apr 9;12:100296. doi: 10.1016/j.jmccpl.2025.100296 (PMC12022632; doi:10.1016/j.jmccpl.2025.100296)
Supplement: Supplementary file 1 — Supplementary figures [file mmc1.pptx]

## Slide 1
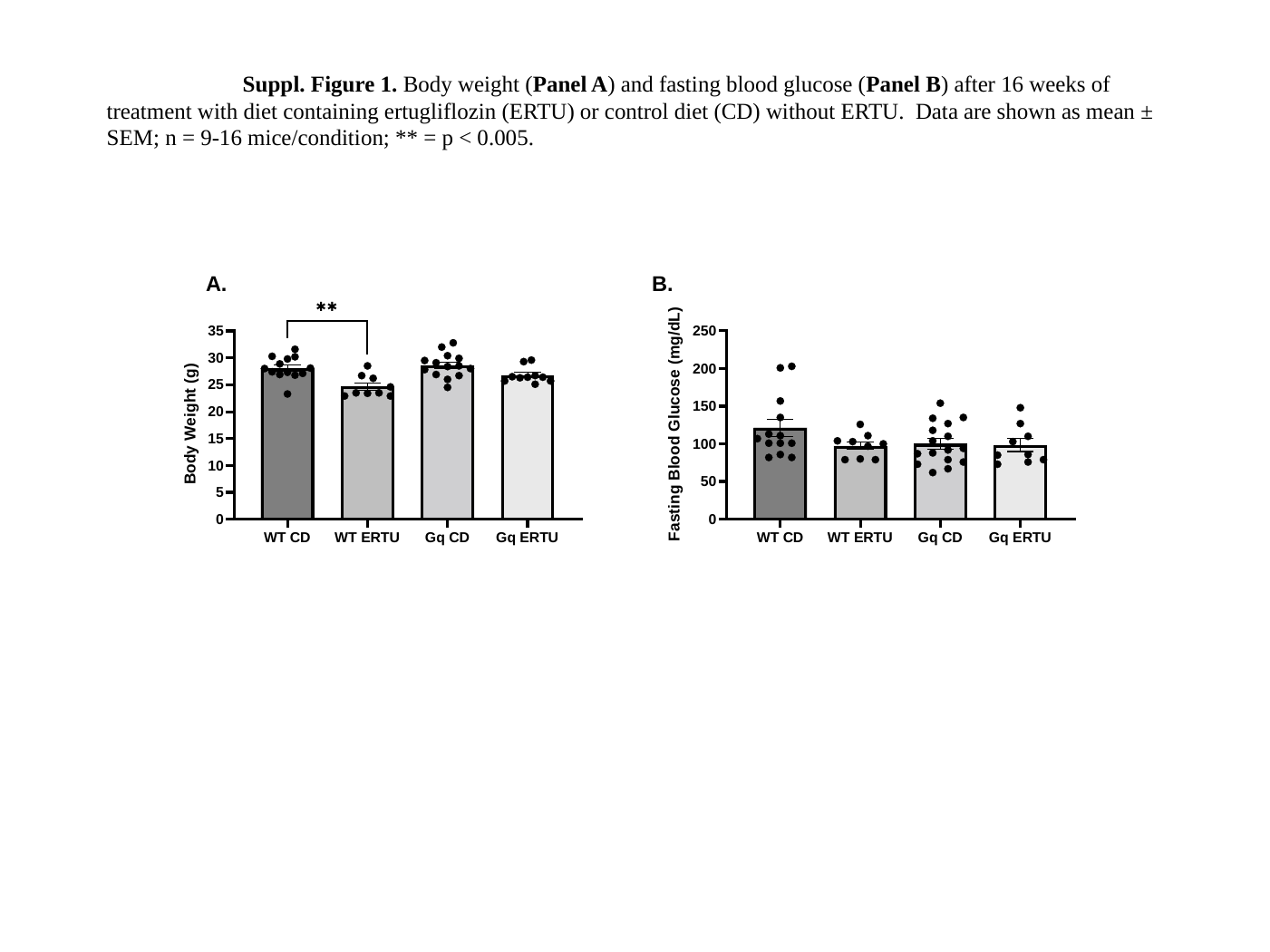

Suppl. Figure 1. Body weight (Panel A) and fasting blood glucose (Panel B) after 16 weeks of treatment with diet containing ertugliflozin (ERTU) or control diet (CD) without ERTU. Data are shown as mean ± SEM; n = 9-16 mice/condition; ** = p < 0.005.

## Slide 2
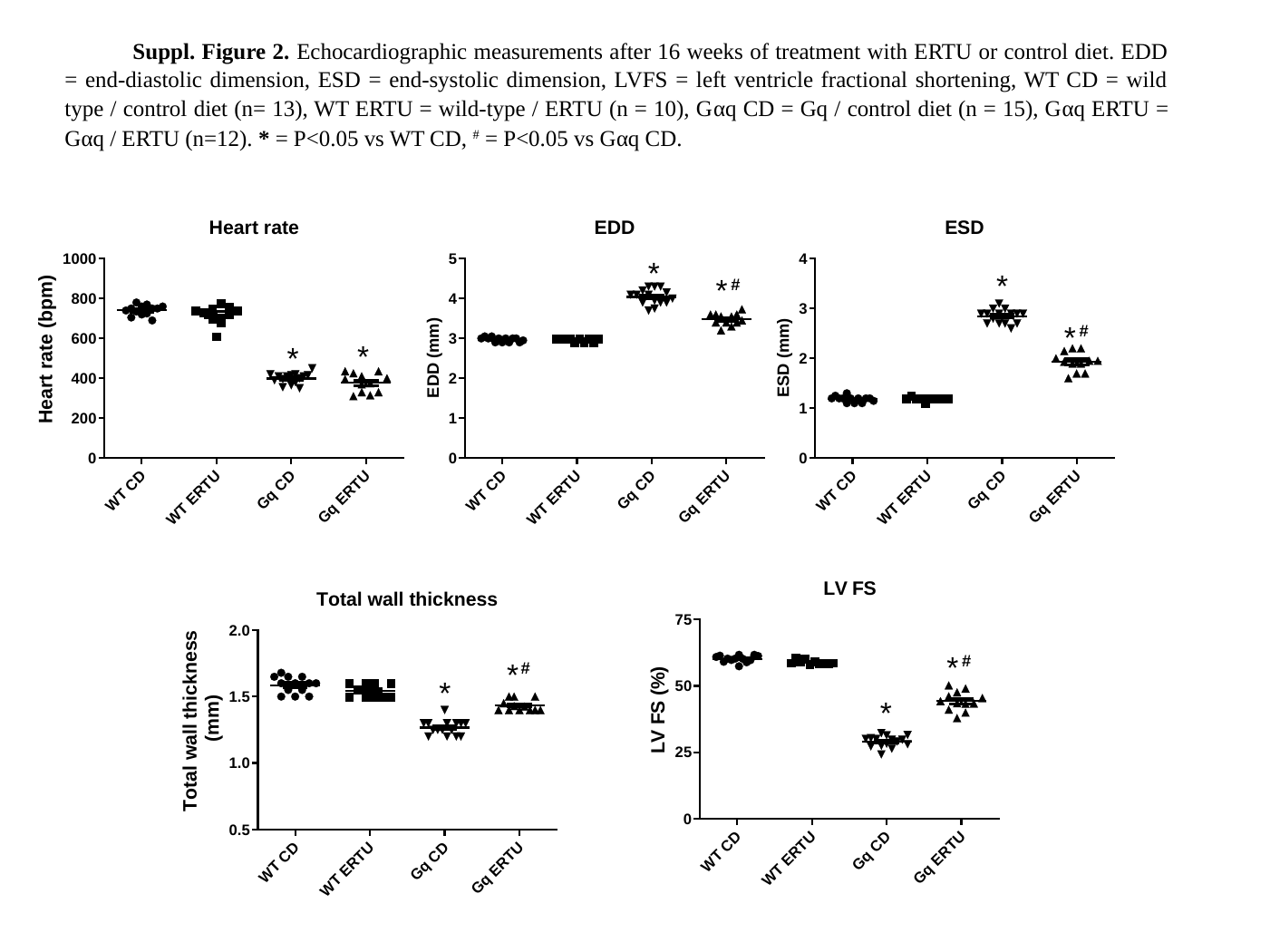

Suppl. Figure 2. Echocardiographic measurements after 16 weeks of treatment with ERTU or control diet. EDD = end-diastolic dimension, ESD = end-systolic dimension, LVFS = left ventricle fractional shortening, WT CD = wild type / control diet (n= 13), WT ERTU = wild-type / ERTU (n = 10), Gαq CD = Gq / control diet (n = 15), Gαq ERTU = Gαq / ERTU (n=12). * = P<0.05 vs WT CD, # = P<0.05 vs Gαq CD.
